# Child feeding practices in rural Ethiopia show increasing consumption of unhealthy foods

**DOI:** 10.1111/mcn.13401

**Published:** 2022-07-19

**Authors:** Woinshet Tizazu, Arnaud Laillou, Kalle Hirvonen, Stanley Chitekwe, Kaleab Baye

**Affiliations:** ^1^ Center for Food Science and Nutrition Addis Ababa University Addis Ababa Ethiopia; ^2^ Nutrition Section, UNICEF Ethiopia Addis Ababa Ethiopia; ^3^ Development Strategy and Governance Division International Food Policy Research Institute Washington District of Columbia USA; ^4^ The United Nations University World Institute for Development Economics Research Helsinki Finland; ^5^ Research Center for Inclusive Development in Africa Addis Ababa Ethiopia

**Keywords:** child feeding, complementary feeding, diet quality, ultra‐processed foods, unhealthy foods

## Abstract

The quality of complementary feeding can have both short‐ and long‐term health impacts by delaying or promoting child growth and establishing taste preferences and feeding behaviours. We aimed to assess the healthy and unhealthy feeding practices of infants and young children in rural Ethiopia. We conducted two rounds of surveys in December 2017/18 in Habru district, North Wello, rural Ethiopia among caregivers of infants and young children (*N* = 574). We characterised the consumption of infants and young children using non‐quantitative 24 h recall and the World Health Organization infant and young child feeding indicators. Sociodemographic characteristics, anthropometry and haemoglobin concentrations were assessed. Breastfeeding was a norm as 82% and 67% were breastfed in the first and second rounds. Between the two rounds, dietary diversity increased from 5% to 17% (*p* < 0.05), but more pronounced increases were observed in the consumption of ultra‐processed food (UPFs). Up to one‐in‐five (22%) of the children consumed UFPs. With an average of only three food groups consumed, the consumption of nutrient‐dense foods like animal source foods, fruits and vegetables was very low particularly among younger children. UPFs are an additional risk factor that contributes to poor quality diets. Behavioural Change Communication interventions, including those in rural areas, should explicitly discourage the consumption of UPFs. Future studies should aim to quantify the amount of UPFs consumed and evaluate how this is associated with diet adequacy and nutritional outcomes.

## Key messages

- Complementary diets in rural Ethiopia are extremely low in diversity.
- Consumption of unhealthy foods is more prevalent than the proportion that meets the minimum dietary diversity.
- Consumption of ultra‐processed foods was not associated with household wealth score.
- Indicators of unhealthy feeding practices should be integrated into nutrition programme monitoring.

## Graphical abstract

Ultra‐processed foods (UPFs) are an additional risk factor that contributes to poor quality diets in rural Ethiopia. Behavioural Change Communication interventions, including those in rural areas, should explicitly discourage the consumption of UPFs.

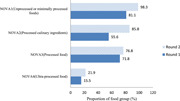

## 1 INTRODUCTION

The world is facing an unprecedented epidemy of malnutrition affecting one in three people (UNICEF/WHO/WB, 2020). While undernutrition has shown some declines, the prevalence of obesity has more than doubled since the 1980s, and is now affecting young children as well (Finucane et al., 2011; Swinburn et al., 2019; WHO, 2021b). In Sub‐Saharan Africa, 36% of children are stunted, but also 5% are overweight (WHO, 2021b). The number of overweight children has almost doubled between 2000 (6.5 million) and 2019 (9.3 million; UNICEF/WHO/WB, 2020). As highlighted by a recent United Nations report, 'there has been no progress to stem the rate of overweight in nearly 20 years in any country‐income group' (UNICEF/WHO/WB, 2020). This lack of progress mirrors the nil, slow, and at times deteriorating trends in diet quality (Baye & Kennedy, 2020).

Diet quality is a shared driver for both forms of malnutrition (Pradeilles et al., 2019). The quality of complementary feeding in particular is critical as it can influence child growth and development, taste and feeding behaviours, all of which can have both short‐ and long‐term impacts (Baye, 2021; Lutter et al., 2021). Traditionally, the focus of infant and young child feeding (IYCF) monitoring in Low and Middle‐Income Countries (LMICs) has been on the nutrient adequacy of complementary foods (WHO, 2003). However, the increasing availability of unhealthy foods, suboptimal feeding practices, and the rising trend in overweight/obesity has led to the recent revision of the WHO IYCF indicators to also capture unhealthy feeding practices (WHO, 2021a).

Unhealthy feeding practices have been on the rise in LMICs, particularly in urban areas (Reardon et al., 2021). A recent study by Nordhagen et al. (2019), assessing the consumption of unhealthy processed foods among children 6–59 months in West Africa, has shown that unhealthy foods were consumed widely (25%–45%), were significantly higher in urban areas, and among older (24–59 months) than younger children (6–23 months). In Nepal, consumption of unhealthy food and beverages among children (12–23 months of age) were associated with lower dietary adequacy and length for age‐z‐score, suggesting that in addition to the direct health impact on the risk of overweight/obesity, such foods can also exacerbate undernutrition (Pries et al., 2019).

However, a recent systematic review was inconclusive on the association of unhealthy foods (i.e., snacks and sugary food and beverages) with child growth or nutrient adequacy of diets, largely due to the variation in measurement and the definition of unhealthy foods/beverages that limited comparability between studies (Pries et al., 2019). However, the IYCF indicator was updated and is now providing a number of agreed definitions/indicators to measure unhealthy feeding practices. In addition, the growing evidence of the negative health impacts associated with the consumption of ultra‐processed foods (UPFs) as measured by the NOVA classification also provides an alternative indicator for assessing unhealthy feeding practices (Chang et al., 2021; Lawrence & Baker, 2019; Monteiro et al., 2021).

Using two rounds of surveys conducted in December 2017/2018, we followed children residing in Habru district, North Wello, rural Ethiopia. The first round, surveyed children 6–18 months of age (*N* = 574), followed by a second visit of the same children a year later (18–30 months; *N* = 535). Our surveys included a non‐quantitative, open‐ended 24 h recall, which allowed us to capture healthy and unhealthy feeding practices according to the most recent IYCF indicators’ definition and the NOVA classification.

## 2 METHODS

### 2.1 Study design, sampling and data collection tools

The study was conducted in Habru woreda, North Wollo zone, Amhara region, Ethiopia. The population is predominantly semi‐subsistence farming, producing sorghum (*Sorghum bicolour*), teff (*Eragrostis tef*), maize (*Zea mays L.*) and pulses as food staples. Commonly grown vegetables in the study area were kale and potato, while fruits like orange, papaya and guava are grown in some areas of the study.

This study was part of a larger study that aimed to evaluate and promote consumption of fresh foods (Hirvonen et al., 2019). The original study had a sample size of 574 households randomly selected from 60 clusters (*got*) found in 12 *kebeles* (smallest administrative units), aided by a full listing of households with children 6–18 months years of age. The inclusion criteria were for the child and the caregiver to be apparently healthy, living in the Habru district for the last 6 months, and with no intention to leave for another year. The first round of data was collected in December 2017, and the same caregiver/children were revisited approximately 12 months later in December 2018. The attrition rate between the first and second rounds was relatively low (6.8%). The majority of the caregivers (85%) were biological mothers, whereas the remaining were mostly grandmothers.

Post hoc power analyses using the openepi tool (https://www.openepi.com/SampleSize/) reveal that our sample size was more than sufficient to estimate the prevalence of UPF consumption for both rounds. A sample size of 468 was sufficient to estimate the prevalence with a 95% confidence level, using the following equation and assumptions:

$$\text{Sample}\;\text{size}\;n=\left[DEFF*Np\left(1-p\right)\right]\;/[(d^{2}/Z_{1-\alpha /2}^{2}*\left(N-1\right)+p*\left(1-p\right)]$$

Population size (for finite population correction factor or fpc; *N*): 1,000,000

Hypothesised % of UPF consumption in the population (*p*): 18.7±5%

Confidence limits as % of 100 (absolute± %; *d*): 5%

### 2.2 Data collection

#### 2.2.1 Socioeconomic, anthropometric and haemoglobin measurements

The length/height and weight of the children were measured in triplicate using standardised techniques, with children wearing light clothing and no shoes. All anthropometric measurements were made by the same person to avoid inter‐examiner errors. Length/height‐for‐age (LAZ), weight‐for‐age (WAZ) and weight‐for‐length (WLZ) Z scores were calculated using the World Health Organization (WHO) multicenter growth reference (De Onis et al., 2006) and ENA 2007 software. Stunting, underweight and wasting were defined, respectively, as LAZ, WAZ or WLZ < −2.

Socio‐demographic characteristics of participants were assessed using a pre‐tested questionnaire that included questions on livelihood activities, education level of parents, health and sanitary facilities, ownership of land and access to electricity. A wealth score (1–10) was computed based on 20 asset‐related variables, using principal component analyses.

Haemoglobin was measured on‐site, using a portable photometer (Hemocue, HB 301). After cleaning the infants’ middle finger with a disinfectant wipe, the side of the finger was pricked using a lancet. After wiping the first two drops, light pressure was applied, and the third drop was collected using a micro‐cuvette and haemoglobin concentrations were read and recorded. Haemoglobin concentrations were adjusted for altitude (1500m above sea level) by subtracting 0.5 (WHO, 2011). Children with moderate and severe forms of anaemia were referred to the nearby health centre for follow‐up.

#### 2.2.2 Food consumption and child feeding practices

##### Minimum dietary diversity

The complementary feeding practices were characterised using WHO's most recently updated IYCF indicators (WHO, 2021a). Food groups consumed in 24 h prior to the survey were captured using an open‐ended food consumption questionnaire. Dietary diversity was calculated using the following eight food groups: (1) grains, roots, and tubers; (2) legumes and nuts; (3) dairy products (milk, yoghurt and cheese); (4) eggs; (5) flesh foods (meat, fish, poultry and liver/organ meats); (6) vitamin A‐rich fruits and vegetables, (7) other fruits and vegetables; and (8) breast milk. The minimum dietary diversity (MDD) was considered met if at least five of the eight food groups were consumed.

#### 2.2.3 Unhealthy feeding practices

The proportion of children that consumed sugar‐sweetened beverages, zero fruit and vegetables and UPFs was calculated. Food and beverages consumed in the last 24 h were categorised into the four NOVA classifications (Monteiro et al., 2019), as follows:

1. NOVA 1—Unprocessed or minimally processed foods (fruit, vegetables, eggs, meat, milk, etc.).
2. NOVA 2—Processed culinary ingredients. These are substances obtained directly from group one foods or from nature, like oils and fats, sugar and salt. They are created by industrial processes such as pressing, centrifuging, refining, extracting or mining, and their use is in the preparation, seasoning and cooking of group one foods.
3. NOVA 3—Processed foods. Food products made by adding salt, sugar or other substance found in group 2 to group 1 foods, using preservation methods such as canning and bottling, and, in the case of breads and cheeses, using non‐alcoholic fermentation. Food processing here aims to increase the durability of group 1 foods and make them more enjoyable by modifying or enhancing their sensory qualities.
4. NOVA4—UPFs. UPFs are formulations of ingredients, mostly of exclusive industrial use, that result from a series of industrial processes.

UPF consumption was captured as a binary outcome, with 1 indicating consumption and 0 no consumption. Supporting Information: Table S2 presents the categorisation of foods into the NOVA classification.

### 2.3 Statistical analyses

The data were cleaned and double‐entered into SPSS (IBM SPSS Statistics 22.0) for statistical analyses. Descriptive statistics of continuous and categorical variables were presented as mean ± SD/median (interquartile range) or in frequency/percentages, respectively.

### 2.4 Ethics

Ethical approval was obtained from the ethical clearance committees of the College of Natural and Computational Sciences, Addis Ababa University, and the Amhara Regional Health Bureau. Consent was obtained from mothers or caregivers, after explaining the purpose and methods of the study. All questionnaires were translated into the local language, Amharic.

## 3 RESULTS

The socio‐demographic characteristics of the study participants (*n* = 574) are summarised in Table 1. Most of the caregivers were married and subsistent farmers (>80%). Of the 574 participants, more than 2/3rd owned their land and were living near to a drinking water supply. The median age of mothers was 27 years, 31% had some formal education, and the majority (96%) were housewives. More than 97% of the participants used firewood for cooking and less than half had pit latrines. Less than 10% had a backyard fruit or vegetable garden. The median (first and third quartiles) age (months) of the children in the first round was 11 (8, 14) and 23 (19, 25) in the second round of the survey (Supporting Information: Table S1).

**Table 1:** Socio‐demographic characteristics of the households selected with infants and young children in North Wello

Selected characteristics	*N* (Frequency in %)
Livelihood (farming)	466 (88.9)
Head of household (father)	443 (77.3)
Husband land ownership	387 (67.5)
Marital status/married	518 (90.7)
Educated mother	175 (30.5)
Caregivers' occupation (housewife)	551 (96.0)
Proportion of first‐born children	206 (35.9)
Sex of children (female)	261 (45.8)
Access to electricity (utility line)	52 (9.2)
Fuel for cooking (wood)	558 (97.2)
Owns the house they currently live‐in	536 (93.5)
Having fruit/vegetable garden	45 (7.8)
Source of drinking water (piped outside compound)	128 (22.3)
Ownership of pit latrine	263 (45.8)

The prevalence of breastfeeding was above 80% in the first round, when most children were in their first year of life; it declined to 67% in the second round as the children got older (Table 2). Between the two rounds, the share of children meeting MDD increased from 5% to 17% (*p* < 0.001), with an average of three food groups consumed (Supporting Information: Table S3). The consumption of grains, roots and tubers, legumes and nuts, and other fruit and vegetables increased over time, but consumption of animal source foods remained very low.

**Table 2:** Food groups consumed by infants and young children (6–30) months in North Wello

	*N* (Proportion in %)	
Food groups	Round 1	Round 2
Breast milk	470 (82.2)	344 (66.9)
Grains, roots and tubers	483 (85.2)	523 (97.9)
Legumes and nuts	346 (61)	435 (81.9)
Dairy products	160 (28.2)	118 (22.1)
Flesh foods	10 (1.8)	15 (2.8)
Eggs	20 (3.5)	18 (3.4)
VA‐rich fruits and vegetables	8 (1.4)	38 (7.1)
Other fruits and vegetables	245 (43.2)	378 (71.1)
Minimum dietary diversity score (≥5 food groups)	29 (5.1)	93 (17.5)
Sugar‐sweetened beverages	6 (1.1)	5 (0.9)
Ultra‐processed food consumption	88 (15.5)	117 (21.9)
Zero vegetable and fruit consumption	319 (56.3)	86 (16.6)

*Note*: VA: vitamin A. The median (Q1, Q3) child age in Round 1 is 11 (8, 14) months and in Round 2 is 23 (19, 25).

**Figure 1:**
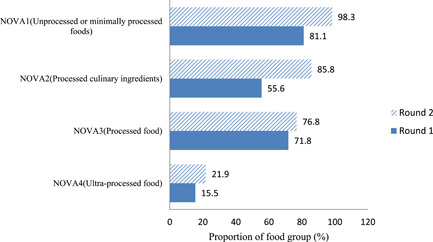
Percentage of NOVA food groups consumed by infants and young children (6–30) months in North Wello

**Figure 2:**
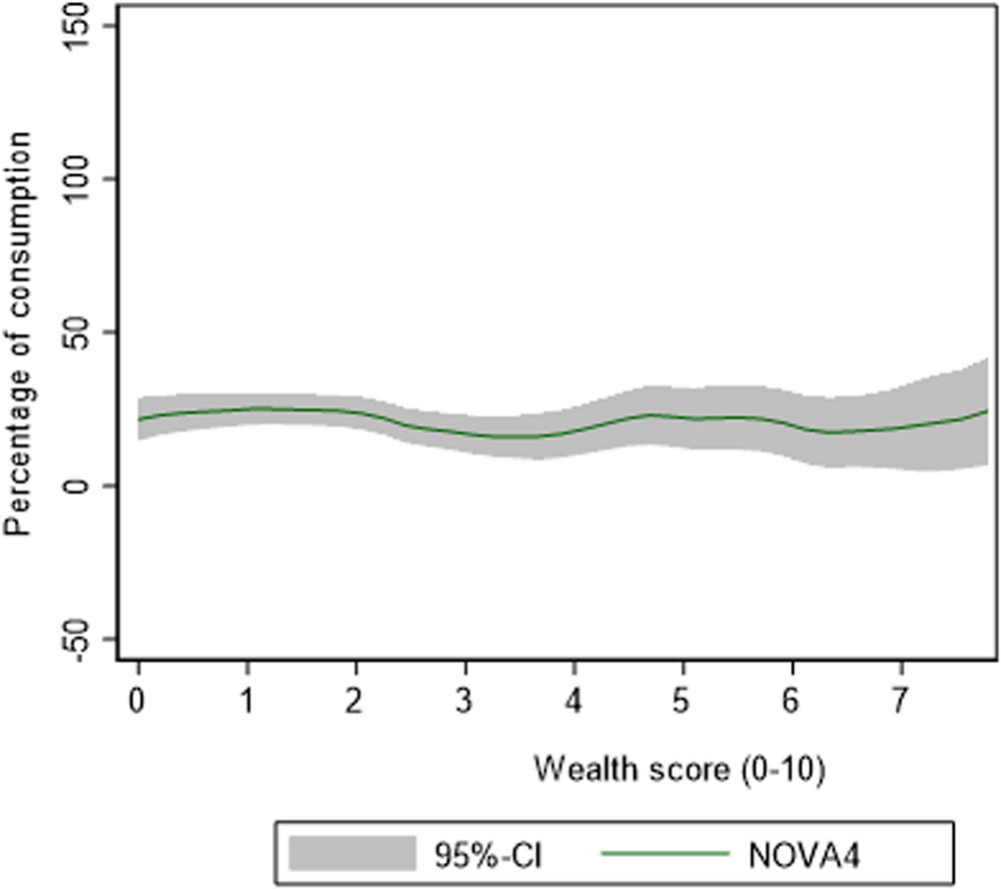
Association between consumption of NOVA4 and wealth score, round 2. *p* Value for the association between NOVA4 consumption and wealth score as tested by a chi‐square was 0.217.

About 15% of children in these rural households consumed UPFs (NOVA4) and this number increased to 22% (*p* < 0.05) in the second round as the children got older (Figure 1). Surprisingly, NOVA4 consumption was not associated with wealth score (Figure 2).

## 4 DISCUSSION

The present study showed that children's diet remained poor in diversity, but more striking is the observation that UPFs have penetrated and are being increasingly consumed by children in rural Ethiopia. Up to a fifth of the children consumed UPFs, a figure that is higher than those that meet the MDD. This illustrates that not only diets remain poor in nutrient adequacy but they are also becoming unhealthy.

Despite the promotion of the numerous benefits of diverse diets, children's diet in Ethiopia remained very low in diversity. Nutrient‐dense foods like fruits, vegetables, and ASFs are rarely consumed. In this study, close to half of the children aged 6–18 months consumed fruits or vegetables in the last 24 h. Although much better for older children (18–30 months), only one in four consumed any fruit or vegetable. ASF consumption was also very low, with less than 5% of children consuming eggs or flesh foods. Such poorly diverse diets, low in nutrient‐dense foods, can expose children to nutrient deficiencies that can compromise their growth and development (Moursi et al., 2008). Poorly diverse diets can also instil unhealthy feeding practices that can persist over time (Lutter et al., 2021). Indeed, the "Fill the Nutrient Gap" analysis implemented in Ethiopia showed that a nutrient‐adequate diet would cost on average 3.8 times more than a diet meeting caloric needs only (WFP/EPHI, 2021). Consequently, only 30% of Ethiopian households could afford the minimum cost nutritious diet (WFP/EPHI, 2021), indicating that households' income needs to be raised, and nutrient‐dense foods be more available and affordable if diets are to be improved (Baye et al., 2020).

Even more alarming is the increasing consumption of UPFs, which introduce unhealthy feeding habits, but also negatively affect the quality of the children's diet by displacing more nutrient‐dense and healthier foods (Pries et al., 2019). Unlike fruit and vegetables and ASFs, whose consumption has been consistently reported to increase with wealth, the association between UPF consumption and wealth in this study was flat and statistically non‐significant (Figure 2). This suggests that the widely held assumption that UPF consumption increases with increased income does not hold in this setting. The limited income that households have in rural Ethiopia could thus be turned away from healthier options to the purchase of cheaper, yet unhealthy processed foods (Gupta et al., 2019). Besides being cheaper, UPFs which are often high in sugar and salt serve as a comforting food for children, are regarded as ʻmodern’, and are also highly appealing for children (Chang et al., 2021).

UPF consumption often associated with greater weight gain could also give a false reassurance to mothers in settings where undernutrition is high and can thus further promote its consumption. This can especially be the case, given that mothers in low‐income settings are often concerned over the poor appetite of their children and want their children to gain weight (Flax et al., 2020). This can make efforts to improve children's diet quality even more difficult to succeed (Gupta et al., 2019). Prospective studies indicated that consumption of UPF among children is associated with added sugar content in the diets and even influences anthropometric and glucose profiles (Costa et al., 2019; Neri et al., 2019). Besides, the development of unhealthy feeding habits through the potentially addictive behaviour of UPF is likely to have long‐term health effects (Filgueiras et al., 2019; Gearhardt & Schulte, 2021). In children, consumption of UPF was associated with cardiometabolic risk and asthma (Elizabeth et al., 2020). There is also a growing evidence on the association between UPF, obesity, and a gut microbiota favouring systemic inflammation and oxidative changes that also lead to reduced cognitive function (Zinöcker & Lindseth, 2018).

The present study has strengths and limitations. First, our findings may not be generalisable to other rural areas in Ethiopia but play an important role of sounding the alarm that UPFs and related unhealthy feeding practices are being picked‐up in rural areas of Ethiopia. Therefore, our findings urge more systematic assessments of unhealthy feeding patterns in Ethiopia. Second, the NOVA classification has in the past been criticised of not being clear and generalisable (Petrus et al., 2021), but growing global literature is revealing the negative health effects associated with UPFs, particularly for foods categorised as NOVA4 (Chang et al., 2021; Lawrence & Baker, 2019). Third, our assessment of the exposure to NOVA4 foods was captured by a binary outcome, instead quantifying the amount (in grams) and the share in energy of NOVA4 foods relative to the overall diet would have been more useful. Fourth, our study remains an observational study evaluating longitudinally children exposed and unexposed to UPF; nevertheless, the study design does not allow to make causal inferences. A strength of our study includes the use of the recently agreed IYCF indicators that include indicators on unhealthy feeding practices. The relatively large sample size, and the longitudinal study design that allowed us to follow children for 1 year at a very critical period of their development (i.e., complementary feeding period) are additional strengths of this study.

Over the last two decades, Ethiopia has been focusing on reducing undernutrition, but these efforts have not considered the consumption of unhealthy foods/UPFs as an additional factor contributing to poor quality diets. Instead, such foods when fortified with nutrients, have been promoted through claims that they can prevent micronutrient deficiencies (Popkin et al., 2021). However, with the recognition of the multiple adverse effects related to the consumption of UPFs, and the need for double‐duty actions that prevent multiple forms of malnutrition, the promotion of UPFs as a vehicle of fortified foods should be restricted. Behavioural Change Communication (BCC) interventions, including those in rural areas, should explicitly discourage the consumption of UPFs; hence, BCC interventions need to be revised to address this changing nutrition reality. Easy‐to‐understand front‐of‐pack labelling giving warnings on the content of unhealthy ingredients as experienced in a growing number of countries may also need to be considered. In addition to MDD, indicators of unhealthy feeding (e.g., zero fruit and vegetable and unhealthy foods) practices should be integrated into nutrition programme monitoring. Future studies should aim to quantify the amount of UPF consumed and how this is associated with diet adequacy and nutritional outcomes.

## AUTHOR CONTRIBUTIONS

Woinshet Tizazu and Kaleab Baye conceived the study. Woinshet Tizazu, Kalle Hirvonen, and Kaleab Baye executed the fieldwork; Woinshet Tizazu, Arnaud Laillou, and Kaleab Baye wrote the paper with inputs from Stanley Chitekwe and Kalle Hirvonen. All authors read and approved the final manuscript.

## CONFLICT OF INTEREST

The authors declare no conflicts of interest.

## Supporting information

Supporting information.

## Data Availability

The data that support the findings of this study are available from the corresponding author upon reasonable request.

## References

[mcn13401-bib-0001] Baye, K. (2022). Improved diet quality, a missing ingredient for accelerating stunting reduction: An example from Ethiopia. Archives of Disease in Childhood, 107(1), 5–6. 33402327 10.1136/archdischild-2020-320292

[mcn13401-bib-0002] Baye, K. , & Kennedy, G. (2020). Estimates of dietary quality in infants and young children (6–23 mo): Evidence from demographic and health surveys of 49 low‐and middle‐income countries. Nutrition, 78, 110875. 32653760 10.1016/j.nut.2020.110875

[mcn13401-bib-0003] Baye, K. , Laillou, A. , & Chitweke, S. (2020). Socio‐economic inequalities in child stunting reduction in Sub‐Saharan Africa. Nutrients, 12(1):253. 10.3390/nu12010253 31963768 PMC7019538

[mcn13401-bib-0004] Chang, K. , Khandpur, N. , Neri, D. , Touvier, M. , Huybrechts, I. , Millett, C. , & Vamos, E. P. (2021). Association between childhood consumption of ultraprocessed food and adiposity trajectories in the Avon longitudinal study of parents and children birth cohort. JAMA Pediatrics, 175(9), e211573. 10.1001/jamapediatrics.2021.1573 34125152 PMC8424476

[mcn13401-bib-0005] Costa, C. S. , Rauber, F. , Leffa, P. S. , Sangalli, C. N. , Campagnolo, P. D. B. , & Vitolo, M. R. (2019). Ultra‐processed food consumption and its effects on anthropometric and glucose profile: A longitudinal study during childhood. Nutrition, Metabolism, and Cardiovascular Diseases, 29(2), 177–184. 10.1016/j.numecd.2018.11.003 30660687

[mcn13401-bib-0006] De Onis, M. , Onyango, A. W. , Borghi, E. , Garza, C. , Yang, H. , & WHO Multicentre Growth Reference Study Group . (2006). Comparison of the World Health Organization (WHO) child growth standards and The National Center for Health Statistics/WHO international growth reference: Implications for child health programmes. Public Health Nutrition, 9(7), 942–947. 17010261 10.1017/phn20062005

[mcn13401-bib-0007] Elizabeth, L. , Machado, P. , Zinöcker, M. , Baker, P. , & Lawrence, M. (2020). Ultra‐processed foods and health outcomes: A narrative review. Nutrients, 12(7), 1955. 32630022 10.3390/nu12071955 PMC7399967

[mcn13401-bib-0008] Filgueiras, A. R. , Pires de Almeida, V. B. , Nogueira, P. C. K. , Domene, S. M. A. , Eduardo da Silva, C. , Sesso, R. , & Sawaya, A. L. (2019). Exploring the consumption of ultra‐processed foods and its association with food addiction in overweight children. Appetite, 135, 137–145. 30439381 10.1016/j.appet.2018.11.005

[mcn13401-bib-0009] Finucane, M. M. , Stevens, G. A. , Cowan, M. J. , Danaei, G. , Lin, J. K. , Paciorek, C. J. , Singh, G. M. , Gutierrez, H. R. , Lu, Y. , & Bahalim, A. N. (2011). National, regional, and global trends in body‐mass index since 1980: Systematic analysis of health examination surveys and epidemiological studies with 960 country‐years and 9·1 million participants. The Lancet, 377(9765), 557–567. 10.1016/S0140-6736(10)62037-5 PMC4472365 21295846

[mcn13401-bib-0010] Flax, V. L. , Thakwalakwa, C. , Phuka, J. C. , & Jaacks, L. M. (2020). Body size preferences and food choice among mothers and children in Malawi. Maternal & Child Nutrition, 16(4), e13024. 32638514 10.1111/mcn.13024 PMC7507496

[mcn13401-bib-0011] Gearhardt, A. N. , & Schulte, E. M. (2021). Is food addictive? A review of the science. Annual Review of Nutrition, 41, 41–410. 10.1146/annurev-nutr-110420-111710 34152831

[mcn13401-bib-0012] Gupta, S. , Hawk, T. , Aggarwal, A. , & Drewnowski, A. (2019). Characterizing ultra‐processed foods by energy density, nutrient density, and cost. Frontiers in Nutrition, 6, 70. 31231655 10.3389/fnut.2019.00070 PMC6558394

[mcn13401-bib-0013] Hirvonen, K. , Tizazu, W. , & Baye, K. (2019). Impact evaluation of WFP's fresh food voucher pilot programme in Ethiopia. Addis Ababa.

[mcn13401-bib-0014] Lawrence, M. A. , & Baker, P. I. (2019). Ultra‐processed food and adverse health outcomes. BMJ, 365, l2289. 31142449 10.1136/bmj.l2289

[mcn13401-bib-0015] Lutter, C. K. , Grummer‐Strawn, L. , & Rogers, L. (2021). Complementary feeding of infants and young children 6 to 23 months of age. Nutrition Reviews, 79, 825–846. 33684940 10.1093/nutrit/nuaa143

[mcn13401-bib-0016] Monteiro, C. A. , Cannon, G. , Levy, R. B. , Moubarac, J.‐C. , Louzada, M. L. C. , Rauber, F. , Khandpur, N. , Cediel, G. , Neri, D. , & Martinez‐Steele, E. (2019). Ultra‐processed foods: What they are and how to identify them. Public Health Nutrition, 22(5), 936–941. 30744710 10.1017/S1368980018003762 PMC10260459

[mcn13401-bib-0017] Monteiro, C. A. , Lawrence, M. , Millett, C. , Nestle, M. , Popkin, B. M. , Scrinis, G. , & Swinburn, B. (2021). The need to reshape global food processing: A call to the United Nations Food Systems Summit. BMJ Global Health, 6(7), e006885. 10.1136/bmjgh-2021-006885 PMC8319974 34321237

[mcn13401-bib-0018] Moursi, M. M. , Arimond, M. , Dewey, K. G. , Treche, S. , Ruel, M. T. , & Delpeuch, F. (2008). Dietary diversity is a good predictor of the micronutrient density of the diet of 6‐to 23‐month‐old children in Madagascar. The Journal of Nutrition, 138(12), 2448–2453. 19022971 10.3945/jn.108.093971

[mcn13401-bib-0019] Neri, D. , Martinez‐Steele, E. , Monteiro, C. A. , & Levy, R. B. (2019). Consumption of ultra‐processed foods and its association with added sugar content in the diets of US children, NHANES 2009‐2014. Pediatric Obesity, 14(12), e12563. 31364315 10.1111/ijpo.12563

[mcn13401-bib-0020] Nordhagen, S. , Pries, A. M. , & Dissieka, R. (2019). Commercial snack food and beverage consumption prevalence among children 6–59 months in West Africa. Nutrients, 11(11), 2715. 31717487 10.3390/nu11112715 PMC6893794

[mcn13401-bib-0021] Petrus, R. R. , José do Amaral Sobral, P. , Tadini, C. C. , & Gonçalves, C. B. (2021). The NOVA Classification System: A critical perspective in food science. Trends in Food Science & Technology, 116, 603–608.

[mcn13401-bib-0022] Popkin, B. M. , Barquera, S. , Corvalan, C. , Hofman, K. J. , Monteiro, C. , Ng, S. W. , Swart, E. C. , & Taillie, L. S. (2021). Towards unified and impactful policies to reduce ultra‐processed food consumption and promote healthier eating. The Lancet Diabetes & Endocrinology, 9(7), 462–470. 10.1016/S2213-8587(21)00078-4 33865500 PMC8217149

[mcn13401-bib-0023] Pradeilles, R. , Baye, K. , & Holdsworth, M. (2019). Addressing malnutrition in low‐ and middle‐income countries with double‐duty actions—ERRATUM. Proceedings of the Nutrition Society, 78, 590. 10.1017/S002966511800280X 30678743

[mcn13401-bib-0024] Pries, A. M. , Filteau, S. , & Ferguson, E. L. (2019). Snack food and beverage consumption and young child nutrition in low‐and middle‐income countries: A systematic review. Maternal & Child Nutrition, 15, e12729. 31225715 10.1111/mcn.12729 PMC6618154

[mcn13401-bib-0025] Pries, A. M. , Rehman, A. M. , Filteau, S. , Sharma, N. , Upadhyay, A. , & Ferguson, E. L. (2019). Unhealthy snack food and beverage consumption is associated with lower dietary adequacy and length‐for‐age z‐scores among 12–23‐month‐olds in Kathmandu Valley, Nepal. The Journal of Nutrition, 149(10), 1843–1851. 31309223 10.1093/jn/nxz140 PMC6768809

[mcn13401-bib-0026] Reardon, T. , Tschirley, D. , Liverpool‐Tasie, L. S. O. , Awokuse, T. , Fanzo, J. , Minten, B. , Vos, R. , Dolislager, M. , Sauer, C. , & Dhar, R. (2021). The processed food revolution in African food systems and the double burden of malnutrition. Global Food Security, 28, 100466. 33868911 10.1016/j.gfs.2020.100466 PMC8049356

[mcn13401-bib-0027] Swinburn, B. A. , Kraak, V. I. , Allender, S. , Atkins, V. J. , Baker, P. I. , Bogard, J. R. , Brinsden, H. , Calvillo, A. , De Schutter, O. , & Devarajan, R. (2019). The global syndemic of obesity, undernutrition, and climate change: The lancet commission report. The Lancet, 393(10173), 791–846. 10.1016/S0140-6736(18)32822-8 30700377

[mcn13401-bib-0028] UNICEF/WHO/WB . (2020). *UNICEF/WHO/The World Bank Group Joint Child Malnutrition Estimates: Levels and trends in child malnutrition: Key findings of the 2020 Edition*.

[mcn13401-bib-0029] WFP/EPHI . (2021). Fill the nutrient gap‐Ethiopia. Addis Ababa.

[mcn13401-bib-0030] WHO . (2003). *Complementary feeding: Report of the global consultation, and summary of guiding principles for complementary feeding of the breastfed child*.

[mcn13401-bib-0031] WHO . (2011). Haemoglobin concentrations for the diagnosis of anaemia and assessment of severity. World Health Organization.

[mcn13401-bib-0032] WHO . (2021a). *Indicators for assessing infant and young child feeding practices: Definitions and measurement methods*.

[mcn13401-bib-0033] WHO . (2021b). *Levels and trends in child malnutrition: UNICEF/WHO/The World Bank Group Joint Child Malnutrition Estimates: Key findings of the 2021 Edition*.

[mcn13401-bib-0034] Zinöcker, M. K. , & Lindseth, I. A. (2018). The western diet–microbiome‐host interaction and its role in metabolic disease. Nutrients, 10(3), 365. 29562591 10.3390/nu10030365 PMC5872783

