# Supplementary material for: Child feeding practices in rural Ethiopia show increasing consumption of unhealthy foods
Source: Matern Child Nutr. 2022 Jul 19;20(Suppl 5):e13401. doi: 10.1111/mcn.13401 (PMC11258765; doi:10.1111/mcn.13401)
Supplement: Supplementary file 1 — Supporting information. [file MCN-20-e13401-s001.docx]

**Supplement**

**Table S1**. Anthropometric status and anemia in infants and young children aged (6 to 30) months in North Wello.

| Variables | Round1 | Round2 |
| --- | --- | --- |
| Median child Age (months) | 11 (8, 14) | 23 (19, 25) |
| Child Weight (kg) | 8.62±1.35 | 10.47±1.39 |
| Child Length (cm) | 72.2±5.14 | 81.30±4.44 |
| HAZ (z-score) | -0.68±1.60 | -1.35±1.53 |
| WAZ (z-score) | -0.56±1.12 | -0.84±1.29 |
| WHZ (z-score) | -0.27±1.26 | -0.28±1.04 |
| Stunted (N(%)) | 96(16.8) | 145(27.3) |
| Underweight (N(%)) | 51(8.9) | 75(14.1) |
| Wasted (N(%)) | 39(6.8) | 34(6.4) |
| Child MUAC (cm) | 14.02±1.02 | 14.19±1.01 |
| Hemoglobin (g/dl)^a^ | 10.49±1.44 | 11.13±1.30 |
| Anemia (N (%)) | 316(57.9) | 166(39.4) |

Note: A threshold used for moderate anemia <11 g/dl anemic, corrected for altitude (1,500 m) based on (WHO, 2011).

**Table S2** Categorization of foods by NOVA

| **NOVA Groups** | **Food groups consumed** |
| --- | --- |
| NOVA1 (Unprocessed or minimally processed foods) | Milk such fresh animal milk; Soup (Clear broth), Solid Foods: porridge (tef, wheat, maize, sorghum); Any gruel (thin or watery porridge made from rice, oats, wheat, or other grains); grains such as rice, barley, wheat, sorghum, millet or other grains; Legumes such as peas, lentils, beans or pulses, Nuts or seeds such as peanuts, groundnuts, sesame, or sunflower seeds and; starchy roots and tubers such as potatoes, sweet potatoes and cassava, Pumpkin, carrot, squash; dark green leafy vegetables (kale, spinach, amaranth leaves); fruits (bananas, apples, citrus fruits): Any meat such as beef, pork, goat or lamb (organ meats, chicken, ducks, or other birds); eggs, tea and drinking water |
| NOVA2 (Processed culinary ingredients) | Oils and butter, sugar, honey, salt, spices, or condiments (processed hot pepper-*berbere*) |
| NOVA3 (Processed foods) | Freshly made unpackaged milk product such as cheese or yogurt; dried fish or shellfish; *Injera* or *kita* (flat bread), freshly made unpackaged breads; smoked meats and fish, canned fish. |
| NOVA4 (Ultra-processed foods)* | Carbonated soft drinks, baby formula milk, fruit juice(-like) drinks, commercially processed fortified food with added sugar and flavors, candies, chocolates, cakes, cookies, or biscuits, chips, crisp, pastries |

*The main forms of NOVA 4 consumed were candies, biscuits, and SSBs. Formula feeding was less than 2%.

**Table S3** Changes in diet, nutritional status and anemia/hemoglobin concentration over time

|  | **Round 1** |  | **Round 2** | **Difference in %**  **[95% CI]** | **P-value for the difference*** |
| --- | --- | --- | --- | --- | --- |
|  | **n (%)** | | |  |  |
| MDD | 27 (5.6) |  | 85 (17.7) | 12.1[8.0,16.2] | <0.001 |
| Stunting | 82 (15.6) |  | 145 (27.6) | 12[7.6,16.4] | <0.002 |
| Wasting | 36 (7) |  | 34 (6.5) | -0.3[-3.2,2.5] | 0.89 |
| Underweight | 47 (8.9) |  | 75 (14.2) | 5.3[2.3, 8.3] | <0.001 |
| Anemia | 234 (57.9) |  | 160 (39.6) | -18.3[-24.6,-12.1] | <0.001 |
| NOVA4 | 84 (16) |  | 116 (22.1) | 6.1[1.2,10.9] | 0.013 |
|  | Mean (SD) | | | Difference in mean [95% CI] | P-value for the difference** |
| Child age in months | 11.4 (3.5) |  | 22.5 (3.7) | 11.1[10.8,11.3] | <0.001 |
| Weight (Kg) | 8.63 (1.34) |  | 10.46 (1.38) | 1.83[1.76, 1.9] | <0.001 |
| Height (cm) | 72.3 (5.02) |  | 81.3 (4.43) | 9 [8.6,9.3] | <0.001 |
| HAZ | -0.66 (1.54) |  | -1.36 (1.53) | -0.7[-0.85, -0.54] | <0.001 |
| WAZ | -0.55 (1.1) |  | -0.84 (1.29) | -0.29(-0.39, -0.2] | <0.001 |
| WHZ | -0.28 (1.26) |  | -0.28 (1.04) | -0.004[-0.11,0.1] | 0.94 |
| MUAC child (mm) | 14.04 (0.98) |  | 14.19 (1.01) | 0.15[0.06,0.24] | 0.002 |
| Hemoglobin in ug/dl | 10.45 (1.42) |  | 11.14 (1.3) | 0.69[0.54,0.85] | <0.001 |

*McNamar’s test for paired proportion; **Paired t-test; MDD, minimum dietary diversity; MUAC, mid-upper arm circumference; HAZ, height-for-age z score; WAZ, weight-for-age z score; WHZ, weight-for-height z score
